# Editorial: Drug metabolism and transport: the Frontier of personalized medicine

**DOI:** 10.3389/fphar.2023.1246827

**Published:** 2023-07-10

**Authors:** Junmin Zhang, Rong Wang, Sofia Azeredo Pereira

**Affiliations:** ^1^ School of Pharmacy and State Key Laboratory of Applied Organic Chemistry, Lanzhou University, Lanzhou, China; ^2^ Department of Pharmacy, The 940th Hospital of Joint Logistic Support Force of PLA, Lanzhou, China; ^3^ Medical School, Universidade Nova de Lisboa, Lisboa, Portugal

**Keywords:** drug metabolism, drug transport, pharmacokinetics, drug-drug interactions, metabolic enzymes, drug transporters, external factors, gut microbiota

Current personalized drug therapy presents many unique challenges (Pristner and Warth, 2020; van Groen et al., 2022). Pharmacokinetic (PK) and pharmacodynamic (PD) investigations of drugs contribute to ensuring the safety and efficacy of clinical use. In particular, understanding PK properties is essential for drug development and precision drug use. Research on drug metabolizing enzymes and transporter proteins in PK assays is advancing rapidly and accurately provides a new understanding of the transcriptional and post-transcriptional regulatory mechanisms that lead to inter-individual variability in drug therapy (Li et al., 2019). Clinical research on drug-drug interactions (DDI) of drug metabolism or transport is also attracting attention (Tornio et al., 2019). In addition, recent advances such as changes in metabolic enzymes and transporter proteins, and gut bacteria with disease progression help standardize personalized drugs and promote rational clinical use (Zhang et al., 2018; Zhang and Wang, 2022).

This Research Topic includes manuscripts covering the studies focusing on PK, excretion, metabolism, and distribution characteristics of drugs, the elucidation of *in vivo* targets of action, metabolites, metabolic enzymes, and pathways of active molecules of herbal medicines based on metabolomics, network pharmacology, and transcriptomics, the influence of transporters, cytochrome P450, metabolite gene polymorphisms on drug metabolism kinetics, the studies of the complex interactions between the gut microbiota and bile acids, all that have a significant impact on the rational drug use.

First, multiple reports focus on the pharmacokinetics, excretion, metabolism, and distribution characteristics of drugs to contribute to clinical translation or rational drug use. He et al. reported the pharmacokinetics, excretion, metabolism, and distribution characteristics of a body-protective compound 157 (BPC157) in rats and dogs. The results revealed that the main excretion pathways of compound BPC157 involved urine and bile. These results contribute to the clinical translation of the target compound. A study by Shen et al., in which 376 steady-state trough concentrations of valproic acid (VPA) were collected from 103 children with epilepsy, aimed to establish a population pharmacokinetic (PPK) model of VPA in children with epilepsy in southern China to provide guidance for individualized dosing of VPA therapy. Fu et al. identified the pharmacokinetics of unbound teicoplanin in Chinese adult patients and proposed an adjusted dosing regimen depending on glomerular filtration rate (eGFR) and serum albumin concentration to serve as a rationale for unconjugated drug-guided dosing. Liu et al. presented the pharmacokinetics and tissue distribution of cryptotanshinone in the treatment of bleomycin-induced idiopathic pulmonary fibrosis in rats. The findings suggest that the pathological changes of pulmonary fibrosis favor the pulmonary exposure of cryptotanshinone and serve as a therapeutic target organ for cryptotanshinone. These results offer new references for pharmacokinetic and pharmacodynamic research on experimental drugs.

Secondly, several works illustrate the *in vivo* targets, metabolites, metabolic enzymes and pathways for the active molecules of herbal medicines using metabolomics, network pharmacology and transcriptomics. Song et al. demonstrated a therapeutic effect of crocin I (CR) on intrahepatic cholestasis (IC) by combined metabolomic and transcriptomic analysis. This therapeutic effect is attributed to the regulation of bile acid and bilirubin biosynthesis in the bile secretion pathway and the expression of 3-hydroxy-3-methylglutaryl-coenzyme A reductase, sulfotransferase 2A1, and ATP-binding cassette transporter G5. Deng et al. elucidated the mechanism of dracorhodin perchlorate topically applied to diabetic foot ulcer (DFU) rats using a combination of metabolomics and network pharmacology. The findings suggest that dracorhodin perchlorate improves wound healing in DFU through multiple targets and pathways and is potentially useful for the treatment of DFU. A study performed by Sun et al. reports the establishment of an “active compound-target-metabolic pathway” network based on an integrated GC-TOF/MS metabolomics and network pharmacology analysis strategy. This approach identified the active compounds, targets and metabolic pathways involved in the Epimedium fried with suet oil to warm kidney and enhance yang. The authors finally revealed that the mechanism of action of Epimedium fried with suet oil to warm kidney and enhance yang is primarily involved in oxidative stress and amino acid metabolism. By analyzing the plasma metabolomics of liver transplant recipients, Zhu et al. identified microbiota-derived uremic retention solutes, bile acids, steroid hormones, and medium- and long-chain acylcarnitines as the major metabolites associated with dose-adjusted trough concentrations of tacrolimus in liver transplant recipients. This work provides a rationale for predicting the optimal dose of tacrolimus to be administered to liver transplant recipients.

Additionally, several research and reviews focused on the effect of transporters, cytochrome P450, and metabolite gene polymorphisms on the pharmacokinetics of drugs. Annisa et al. reviewed the effect of transporter and metabolite gene polymorphisms on the kinetic parameters of fluoroquinolone, suggesting that the presence of gene polymorphisms can affect the pharmacokinetic parameters of fluoroquinolone such as area under the curve (AUC), creatinine clearance (C_Cr_), maximum plasma concentration (C_max_), half-life (t_1/2_), and time to peak (t_max_). Zhu et al. revealed a novel mechanism by which testis-specific Y-coding-like protein 1 (TSPYL1) regulates the expression of cholesterol metabolizing cytochrome P450s (CYPs), especially CYP1B1, through Wnt/β-catenin signaling. Their results increase the possibility that TSPYL 1 might be a molecular target affecting cholesterol homeostasis. Xin et al. established a combined population pharmacokinetic (PPK) model of aripiprazole (ARI) and its main active metabolite dehydroaripipiprazole (DARI) in pediatric patients with tic disorders (TD) and investigated inter-individual variability in ARI pharmacokinetics/pharmacodynamics caused by physiological and genetic factors to optimize dosing regimens in pediatric patients. The findings showed that the pharmacokinetics of ARI and DARI in pediatric patients with TD were significantly influenced by body weight and CYP2D6 genotype. The authors recommend an individualized dosing regimen for pediatric patients with TD to ensure clinical efficacy. Sato et al. examined the relationship between methotrexate (MTX) accumulation and gene expression levels of solute carrier (SLC) and ATP-binding cassette (ABC) transporters in cancer cells after single and high doses of X-ray irradiation. Their results indicate that single high-dose irradiation alters the accumulation of MTX and the gene expression levels of SLC and ABC transporters in cancer cells. Li et al. investigated the metabolic stability and enzymatic kinetics of carboxylesterase-mediated hydrolysis and cytochrome P450 (CYP)-mediated oxidation of Eupalinolide A (EA; Z-configuration) and Eupalinoide B (EB; E-configuration) in human liver microsomes. The results revealed that EA and EB were promptly eliminated compared to CYP-mediated oxidation, largely through carboxylesterase-mediated hydrolysis. EA and EB were metabolized by multiple CYPs, with CYP3A4 playing a particularly important role. The findings from Wang et al. revealed that Myriocin enhances the antifungal activity of fluconazole by blocking the membrane localization of the efflux pump Cdr1. Another study by Jiang et al. identified two functional polymorphisms in the ABCC4 gene that may affect transcriptional activity and thus directly or indirectly affect the release of atorvastatin and its metabolites from hepatocytes into the circulation. These results provide a strong rationale for the clinical use of drugs.

There are also two more interesting studies on the combined use of herbal ingredients and Chinese compound prescription. A study by Chen et al. reported on the evaluation and clinical significance of the interaction of compound Danshen dropping pill (CDDP) with epoxide hydrolase gene-related warfarin. The findings indicated that a reasonable combination of CDDP and warfarin is safe without risk of bleeding, but also requires therapeutic management. In another study, Lin et al. reported the effect of ethyl acetate extract (SDEA extract) from *Selaginella doederleinii* hieron on human cytochrome P450 enzymes (CYP450). The results revealed that amentolavone may be one of the main reasons for the inhibition of CYPs by SDEA. When SDEA or amentolavane is used in combination with other clinical drugs, potential herbal interactions should be considered.

In addition, the findings from Malinska et al. that the hypolipidemic effect of salsalate was implicated in the differential expression of genes regulating lipid metabolism in the liver, indicating that the administration of salsalate in prediabetic patients with symptoms of non-alcoholic fatty liver disease (NAFLD) may be beneficial. Zhang et al. reviewed the therapeutic prospects of N-acetylgalactosamine-siRNA couples, highlighting the efficiency and stability of GalNAc-siRNA couples. Their results imply that new approaches to developing oligonucleotide drugs hold promise for gene therapy.

Even though pharmacokinetics, pharmacogenetics and pharmacogenomics have been at the forefront of research aimed at finding new personalized therapies, the focus of recent research has been extended to the potential of the gut microbiota to affect drug efficacy. The complex interactions of the gut microbiota with bile acids may have a significant impact on the pharmacokinetics of drugs. Đanić et al.'s findings suggest that bioaccumulation and biotransformation of simvastatin through gut bacteria may be a potential mechanism for its altered bioavailability and therapeutic efficacy. Li et al. utilized an integrated metabolomics, culturomics and bionomics framework to discover that multiple drugs are metabolized by selected probiotic bacteria, providing practical guidance for probiotic drug combinations and new insights into precision probiotics.

Altogether, the focused release of these results provides sufficient reference data to standardize personalized drugs and promote rational clinical use. We hope you enjoy them.
